# Antifungal activity of cinnamaldehyde against *Aspergillus fumigatus* involves disruption of the TCA cycle and protein metabolism

**DOI:** 10.3389/fmicb.2025.1613987

**Published:** 2025-08-22

**Authors:** Fang Li, Xi Cheng, Ling Li, Jinglu Jiang, Yan Liu, Siyu Mo, Wenxia Jiang, Li Liu, Salem Baldi, Nanbiao Long

**Affiliations:** Department of Medical Laboratory Diagnostics, School of Medical Technology, Shaoyang University, Shaoyang, China

**Keywords:** *Aspergillus fumigatus*, cinnamaldehyde, tricarboxylic acid (TCA) cycle, protein metabolism, itraconazole-resistant strains

## Abstract

*Aspergillus fumigatus* is an environmental opportunistic fungal pathogen, which can lead to invasive aspergillosis in immunocompromised individuals, and resistant to conventional antifungual agents has become a growing concern. This study investigated the antifungal activity and the molecular antifungal mechanisms of Cinnamaldehyde (CA) against *A. fumigatus*, specifically its impact on metabolic pathways and protein metabolism. In susceptibility tests, CA was found to exhibit promising antifungal activity against *A. fumigatus* in both solid and liquid culture (biomass) systems, with the minimum inhibitory concentration (MIC) determined as 40–80 μg/mL. Quantitative spore viability assays under elevated CA concentrations demonstrated that the antifungal efficacy of CA against *A. fumigatus* is primarily attributable to its direct fungicidal mechanism. Interestingly, CA also showed equivalent antifungal activity against itraconazole- resistant strains R1 (ITZ, MIC 8 μg/mL) and R2 (ITZ, MIC 8 μg/mL), as it did against its parental strain Af293 (ITZ, MIC 1.5 μg/mL), suggesting its potential value to overcome resistance mechanisms associated with conventional antifungal therapies. Further proteomics and metabolomics analyses revealed that CA significantly affected the tricarboxylic acid (TCA) cycle and protein metabolism, with 167 differentially expressed proteins and 350 altered metabolites identified after 180 min of treatment (FC > 2 or <0.5, *p* < 0.05, VIP > 1). Following treatment with CA, the protein expression of the putative translation initiation factor eIF4E3 (AFUB_051690), the putative leucyl-tRNA synthetase LeuRS (AFUB_093380), prolyl-tRNA synthetase ProRS (AFUB_010170) and the putative peptidyl-tRNA hydrolase Pth1 (AFUB_053480) exhibited a significant decrease. Moreover, deletion of *pth1* resulted in a severe growth defect and hypersensitivity to CA, as evidenced by complete growth arrest at 30 and 45 μg/mL CA. Altogether, the results uncovered a novel antifungal mechanism of CA against *A. fumigatus* and suggest that CA or its derivatives could be developed as effective antifungal drugs.

## Introduction

Invasive fungal infections present a formidable global public health challenge, with an estimated annual toll of 1.5 to 2 million deaths (Gupta et al., 2021). More than 600 fungal species have been recognized as being capable of instigating infections in humans. Among this diverse array, *Aspergillus* spp. account for a staggering 70% of the deaths associated with fungal infections (Earle et al., 2023). *A. fumigatus*, a saprophytic mold with a global distribution, can precipitate life-threatening infections in immunocompromised individuals (Langfeldt et al., 2022). Triazole antifungal drugs, principally itraconazole and voriconazole, along with amphotericin B, constitute the frontline medications for treating invasive aspergillosis (IA) (Patterson et al., 2016). However, the increasing prevalence of resistance to these antifungals, especially in *A. fumigatus*, has become a matter of mounting concern (Wiederhold and Verweij, 2020; Rivelli Zea and Toyotome, 2022). The most extensively studied molecular mechanisms underlying azole resistance in *A. fumigatus* primarily include: (i) over-expression of Cyp51A or structural modifications in the Cyp51A protein, (ii) up-regulation of efflux pumps, particularly those belonging to the ATP-binding cassette (ABC) and major facilitator superfamily (MFS) transporter families, and (iii) additional mechanisms involving biofilm formation, cellular stress responses, and potential alterations in sterol metabolism (Perez-Cantero et al., 2020). The emergence of drug resistance in *A. fumigatus* not only throws down new gauntlets to traditional treatment modalities but also sets forth novel requisites for the research and development of novel antifungal agents.

Cinnamaldehyde (CA), which is the principal component of cinnamon essential oil obtained from *Cinnamomum cassia* and *Cinnamomum verum*, is widely utilized as a food additive in industrial products and has been designated as safe (GRAS) by the United States Food and Drug Administration (FDA) (Hajinejad et al., 2020; Usai and Di Sotto, 2023). Numerous studies have shown that CA possesses extensive antibacterial, yeast, and filamentous mold activities (Doyle and Stephens, 2019). Besides, CA also has multiple pharmacological activities, such as anticancer/antitumour (Peng et al., 2024), antioxidant, anti - inflammatory, neuroprotective, and cardioprotective effects (Hariri and Ghiasvand, 2016; Luan et al., 2022; Guo et al., 2024).

The molecular mechanisms through which CA inhibits the growth of fungi are highly intricate, principally encompassing the inhibition of ATPase activity, the suppression of cell wall or biofilm formation, as well as the alteration of the structure and integrity of cell membranes (Shreaz et al., 2016). In *Fusarium sambucinum*, CA inhibits ergosterol biosynthesis to disrupt cell membrane integrity, exhibiting strong antifungal activity (Wei et al., 2020). In *Zygosaccharomyces rouxii*, CA induces apoptosis via a metacaspase- dependent mitochondrial pathway (Wang et al., 2022), while *Aspergillus niger* studies show malate dehydrogenase is its target protein (Wang et al., 2024). Notably, CA treatment in immunosuppressed mice with invasive pulmonary candidiasis enhances fungal clearance and reduces (1,3)-*β*-D-glucan levels compared to fluconazole (Deng et al., 2021).

The proteomic and metabolomic technologies enable comprehensive analysis of fungi responses to drug treatment, overcoming the limitations of single-target studies. Specifically, it can reveal pathway-level perturbations, compensatory mechanisms, and side targets in resistant strains (Gonzalez-Covarrubias et al., 2022; Sulaiman and Lam, 2022). To further investigate the molecular mechanisms underlying the antifungal activity of CA, this study selected *A. fumigatus* as the target organism and implemented proteomics and metabolomics analysis under the treatment of CA. The results demonstrated that the growth inhibition of *A. fumigatus* by CA is intimately associated with the suppression of the TCA cycle and protein metabolism as novel targets beyond ergosterol biosynthesis.

## Materials and methods

### Strains and media

The parental strain of *A. fumigatus* employed in this study was A1160 and Af293 (FGSC). Another strain, A1160^C^, which was labeled as WT in the text, was constructed by reintroducing the pyrG gene into A1160 (Jiang et al., 2014). Itraconazole-resistant strains R1 and R2 were mutated from Af293. For *A. fumigatus* cultivation, YAG (containing 2% glucose, trace elements, 0.5% yeast extract and 2% agar) and YUU (YAG+5 mM uridine, 10 mM uracil) were used. For colony growth tests, *A. fumigatus* strains were grown on YAG or YUU supplemented with the indicated reagent.

### CA susceptibility test and biomass analysis

To evaluate CA’s antifungal activity, YAG plates containing 0, 30, 45, or 60 μg/mL CA were inoculated with 3 × 10^4^ *A. fumigatus* WT spores (A1160 background). After incubation at 37 °C for 2.5 d, colonies were photographed and their diameters measured. Experiments were performed in triplicate for each CA concentration, with growth inhibition assessed by calculating mean colony diameters (±SD).

To assess the antifungal activity of CA against *A. fumigatus* strain Af293 and its drug-resistant variants R1 and R2, 3 × 10^4^, 3 × 10^3^, and 3 × 10^2^ spores were plated on YAG agar containing CA at concentrations of 0, 30 and 45 μg/mL, respectively. After 48-h incubation at 37 °C, colony formation was evaluated to determine growth inhibition.

Biomass quantification was conducted by introducing 5 × 10^7^ *A. fumigatus* WT spores into 100 mL YAG liquid medium containing 30, 40, or 60 μg/mL CA. Biomass was harvested after 20 h of cultivation using sterile gauze filtration and oven-dried at 80 °C to a constant weight. Each sample was assayed in triplicate to ensure statistical reliability.

### Fungicidal assay

To elucidate whether CA exhibits fungistatic or fungicidal activity, a quantitative viability assay was performed. Initially, 1 × 10^8^ *A. fumigatus* WT spores were inoculated into YAG liquid medium. The experimental groups were treated with 200 and 400 μg/mL of CA for 3 h, while the control group remained untreated. Post-incubation, samples were centrifuged at 8000 rpm for 3 min to pellet the spores and remove the CA-containing supernatant. The pelleted spores were then washed twice with sterile water to eliminate residual CA, followed by serial dilution for spread plating. Specifically, the experimental samples were diluted 10^3^-fold, and the control samples were diluted 10^5^-fold to ensure countable colonies. Each treatment group was replicated three times, and colony-forming units (CFUs) were enumerated to statistically assess the impact of CA on fungal viability.

### E-test and MIC assay

For E-test susceptibility analysis, *A. fumigatus* WT spores (4 × 10^6^) were uniformly suspended in 20 mL of molten agar medium, achieving a final concentration of 2 × 10^5^ spores/mL. Following solidification, ITZ E-test strips were aseptically placed at the center of each plate. After a 24-h incubation at 37 °C, drug resistance was assessed by determining the inhibition zone.

For the minimum inhibitory concentration (MIC) assay, the procedure was performed according to the EUCAST DEFINITIVE DOCUMENT E. DEF 9.3. In brief, double-strength RPMI 2% G medium buffered with MOPS was prepared. Serial dilutions of cinnamaldehyde (CA) were prepared to achieve final concentrations of 80, 40, 20, 10 and 0 μg/mL in the test wells, with 1 × RPMI glucose medium serving as the negative control. Each well was inoculated with 2 × 10^5^ *A. fumigatus* WT spores/mL and incubated at 37 °C for 48 h. The MIC was defined as the lowest CA concentration that completely inhibited visible fungal growth after the incubation.

### Ergosterol extraction and quantification

The ergosterol extraction protocol was performed as previously described (Song et al., 2016). Briefly, 5 × 10^7^ WT spores were cultured in 100 mL YAG liquid medium supplemented with or without 45 μg/mL CA at 37 °C with shaking (220 rpm) for 24 h. The harvested mycelia were washed with distilled water, lyophilized, and ground to a powder. For each sample, 100 mg of mycelial powder was saponified with 3 mL of 25% KOH alcoholic solution (methanol: ethanol, 3:2, v/v) at 85 °C for 1 h. The mixture was then extracted with 3 mL of n-pentane. The upper organic phase was collected, evaporated to dryness, and redissolved in 2 mL methanol. The solution was filtered (0.45 μm) before HPLC analysis using a C18 column with 100% methanol mobile phase (flow rate 1 mL/min) and detection at 282 nm.

### Construction of gene deletion mutant and complementation strain

To knock out *pth1* in *A. fumigatus*, the method of homologous recombination was adopted in this study. Firstly, the fragments of approximately 1.5 kb upstream and downstream of the *pth1* gene were amplified by primer pairs pth1 P1/3 and pth1 P4/6, respectively. Subsequently, the screening marker hph fragment was amplified using the primer pair hygF/R. Finally, the three fragments were homologously recombined into the vector pBARGPE1 at the *ClaI* cloning site using pEASY®-Basic Seamless Cloning and Assembly Kit (TransGen Biotech, Beijing). The fused fragment, amplified by the primer pair pth1 P2/5, was introduced into the protoplasts of A1160, yielding the *pth1* transformants. Subsequently, verification of these transformants was carried out using the primer pair pth1 S1/2, which culminated in the successful acquisition of the *pth1* deletion mutant.

For genetic complementation, the *pth1* gene fragment, including its upstream and downstream flanking regions, was amplified using primers pth1com F and pth1com R. The PCR product was then cloned into the *SpeI* site of the pZero-uu vector, which carries the *NcpyrG* selectable marker. The recombinant plasmid was subsequently transformed into protoplasts of the *Δpth1* knockout strain via polyethylene glycol (PEG)-mediated transformation. Transformants were selected on medium lacking uridine/uracil. The oligonucleotides used in this study are displayed in Supplementary Table 1.

### Metabonomic analysis

Metabonomic experiments were performed by Shanghai Applied Protein Technology Co., Ltd. Briefly, the conidia of *A. fumigatus* of the WT strains were inoculated into YAG liquid medium and incubated on a rotary shaker at 220 rpm and 37 °C for 20 h. Six biological replicates were exposed to 75 μg/mL CA for 0, 90 and 180 min, respectively. The samples were then filtered through gauze, rinsed with deionized water, and promptly frozen in liquid nitrogen. For metabolite extraction, the samples were homogenized into a powdered form using liquid nitrogen. For each sample, 80 mg was added to 1 mL of a cold extraction solvent mixture of methanol/acetonitrile/H₂O (2:2:1, v/v/v), followed by thorough vortexing and incubation on ice for 20 min. Subsequently, the samples were centrifuged at 13,000 g for 20 min at 4 °C. The supernatant was collected and passed through a 96-well protein precipitation plate. The elution was collected and dried in a vacuum centrifuge at 4 °C. For LC–MS analysis, samples were redissolved in 100 μL of 1:1 (v/v) acetonitrile/water and analyzed using a Sciex TripleTOF 6,600 quadrupole time-of-flight mass spectrometer coupled to hydrophilic interaction chromatography (HILIC) via electrospray ionization (ESI). Chromatographic separation was performed on an ACQUITY UPLC BEH Amide column (2.1 mm × 100 mm, 1.7 μm; Waters, Ireland) using Solvent A (25 mM ammonium acetate and 25 mM ammonium hydroxide in water) and Solvent B (acetonitrile). The gradient profile was as follows: 85% B for 1 min, linearly reduced to 65% over 11 min, further reduced to 40% in 0.1 min and held for 4 min, then increased back to 85% in 0.1 min, followed by a 5-min re-equilibration period. Flow rate: 0.4 mL/min; column temperature: 25 °C; injection volume: 2 μL. The mass spectrometer was operated in both positive and negative ionization modes. Raw MS data (wiff.scan files) were converted to MzXML format using ProteoWizard MSConvert prior to import into the open-source XCMS software. For peak picking: centWave m/z = 25 ppm, peakwidth = c (10, 60), prefilter = c (10, 100). For peak grouping: bw = 5, mzwid = 0.025, minfrac = 0.5. Differentially expressed metabolites (DEMs) were identified using FC > 2 or <0.5, *p* < 0.05 and VIP > 1 as cutoffs.

### Proteomic analysis

Proteomic experiments were conducted by Shanghai Applied Protein Technology Co., Ltd. The strain cultivation and CA treatment procedures were identical to those of the aforementioned metabolomics treatment methods. A total of three biological replicates were performed for the proteomics analysis. Proteins were extracted using SDT buffer (4% SDS, 100 mM Tris–HCl, 1 mM DTT, pH 7.6) and quantified using the BCA Protein Assay Kit (Bio-Rad, USA). 20 μg of protein per sample were separated by SDS-PAGE, and protein bands were visualized with Coomassie Blue R-250 staining. SDS-PAGE analysis confirmed no evidence of protein degradation, and the protein yield was deemed sufficient for subsequent experiments, thus enabling the progression to formal assays. Subsequently, proteins were digested with trypsin. LC–MS analysis was carried out on a timsTOF Pro mass spectrometer (Bruker) coupled to a Nanoelute (Bruker Daltonics). Digested *A. fumigatus* peptides were loaded onto a reversed-phase trap column (Thermo Scientific Acclaim PepMap100, 100 μm × 2 cm, nanoViper C18) connected to a reversed-phase C18 analytical column (Thermo Scientific Easy Column, 10 cm × 75 μm, 3 μm C18 resin). The peptides were equilibrated in Buffer A (0.1% formic acid) and separated using a linear gradient of Buffer B (84% acetonitrile, 0.1% formic acid) at a flow rate of 300 nL/min, controlled by IntelliFlow technology. The MS raw data of each sample were analyzed using MaxQuant 1.6.14 software against the *A. fumigatus* UniProt database for identification and quantification. FDR was set to 1% at both peptide and protein levels. Differentially expressed proteins (DEPs) were identified using FC > 2 or <0.5 and *p* < 0.05 as cutoffs.

### RNA extraction and qPCR analysis

Fungal cultures were initiated by inoculating 5 × 10^7^ WT spores into YAG medium incubating for 18 h at 37 °C, 220 rpm. Experimental groups were treated with 75 μg/mL CA for either 15, 30 or 60 min, with three biological replicates per treatment condition. Mycelia were harvested by filtration through sterile gauze, washed three times with deionized water, blotted dry, and flash-frozen in liquid nitrogen. For RNA extraction, frozen mycelia were ground in liquid nitrogen, and 100 mg of mycelial powder was added to 1 mL TRIzol reagent. After vortexing and incubation for 5 min at room temperature, 200 μL chloroform was added, followed by vigorous vortexing for 30 s and an additional 5 min incubation. The mixture was centrifuged at 13,000 rpm for 10 min at 4 °C, and 400 μL of the aqueous phase was carefully transferred to a new RNase-free tube. RNA was precipitated by adding an equal volume of isopropanol, incubating for 5 min, and centrifuging at 13,000 rpm for 5 min. The resulting RNA pellet was washed with 75% ethanol, air-dried, and finally dissolved in 300 μL DEPC-treated water. RNA integrity was confirmed by agarose gel electrophoresis. For cDNA synthesis, 0.8 μg of total RNA was reverse transcribed, and the resulting cDNA was diluted fivefold for subsequent qPCR analysis.

## Results

### CA exhibits strong inhibitory activity against *A. fumigatus*

**Figure 1:**
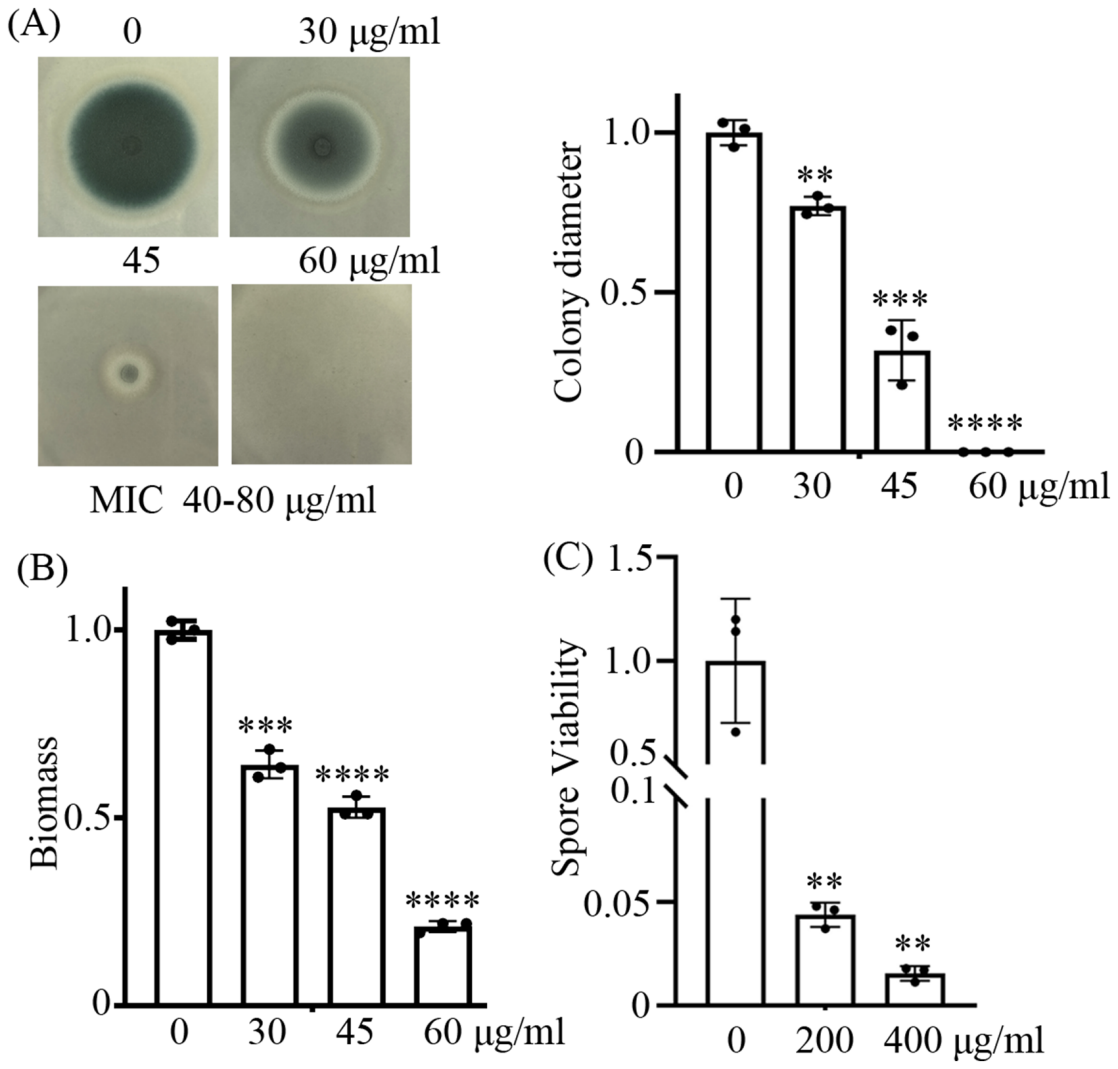
CA shows strong inhibitory capabilities against *A. fumigatus*. **(A)** represents the growth phenotypes of the wild-type strain on YAG solid medium containing 30, 40, and 60 μg/mL CA. Spores were inoculated at a quantity of 3 × 10^4^; **(B)** shows the biomass quantification of *A. fumigatus* in liquid YAG medium treated with 30, 45, and 60 μg/mL CA for 24 h. **(C)** shows the survival rates of spores treated with 200 and 400 μg/mL CA for 3 h. **, ***, and **** indicate statistical significance at *p* < 0.01, *p* < 0.001, and *p* < 0.0001, respectively.

Previous studies have demonstrated that CA possesses potent antifungal activity (Shreaz et al., 2016; OuYang et al., 2019; Long et al., 2024). In this study, we have tested its antifungal activity on the wild-type strain of *A. fumigatus*. Our antifungal assays demonstrated that cinnamaldehyde (CA) exhibits concentration-dependent anti-fungal activity against *A. fumigatus*. In the solid YAG medium supplemented with 30, 40, and 60 μg/mL CA, the mycelial growth of *A. fumigatus* was progressively inhibited. At 30 μg/mL, partial inhibition (23% reduction in colony diameter vs. control) was observed, while 45 μg/mL caused significant suppression (68% reduction). When the concentration was increased to 60 μg/mL, the growth of mycelium was completely inhibited (Figure 1A). Minimum inhibitory concentration (MIC) assays confirmed that the MIC of CA against the WT strain fell within the range of 40–80 μg/mL. Similarly, in the liquid medium, the biomass of *A. fumigatus* decreased in a dose-dependent manner with increasing concentration of CA (Figure 1B), which further confirmed the antifungal potential of CA. To validate whether CA exerts fungistatic or fungicidal effects, *A. fumigatus* spores were exposed to escalating concentrations of CA (200 and 400 μg/mL), followed by quantitative colony-forming unit (CFU) enumeration. Statistical analysis revealed a dose-dependent reduction in CFUs, relative to the untreated control (Figure 1C), strongly indicate that CA exhibits fungicidal activity against *A. fumigatus*. These data indicate that CA shows good antifungal activity against *A. fumigatus*.

### CA exhibited antifungal activity against itraconazole-resistant strains

**Figure 2:**
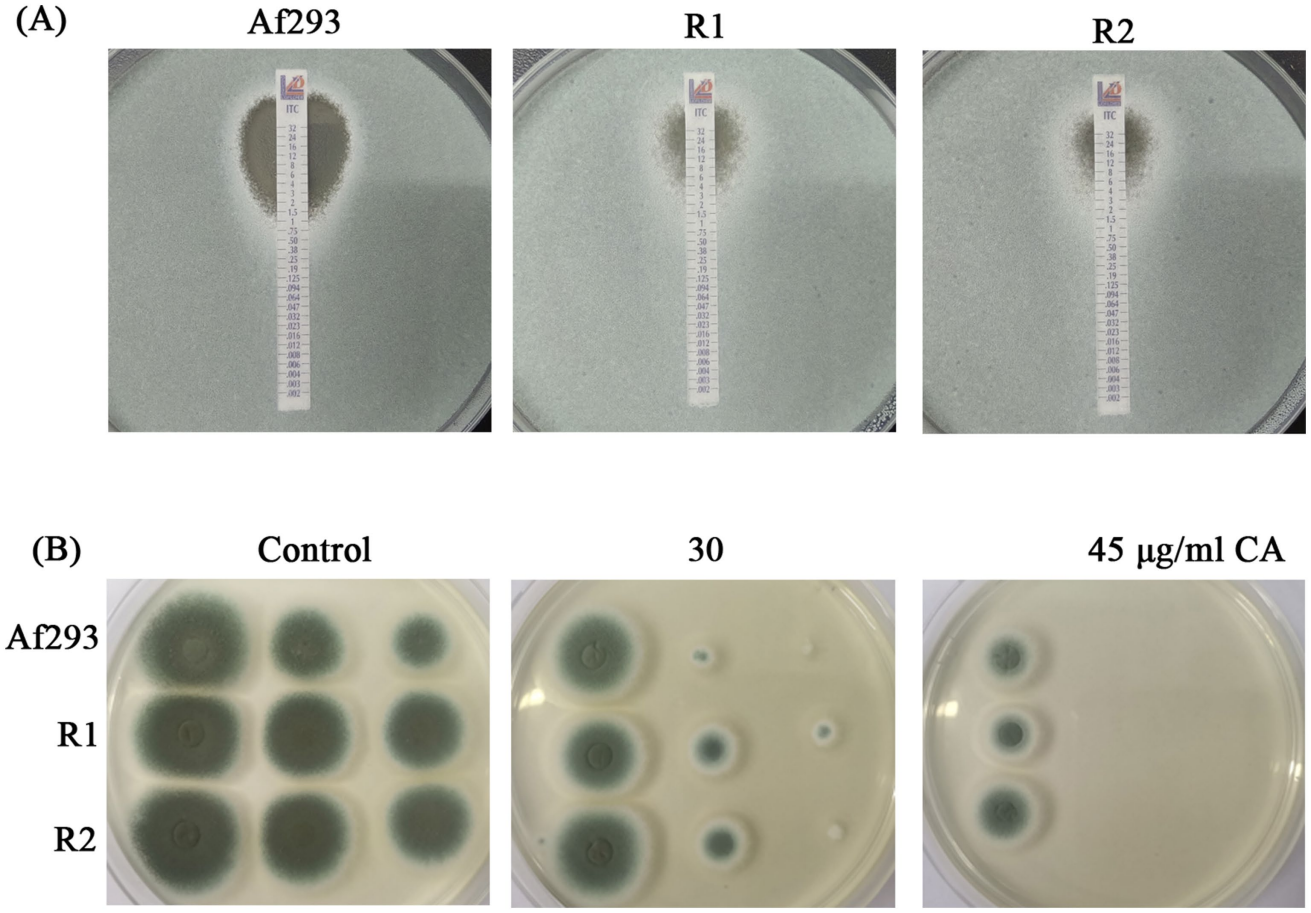
Antifungal activity of CA against itraconazole-resistant strains. **(A)** E-test of Af293, R1 and R2 to ITZ strip. **(B)** CA against itraconazole-resistant strains.

Previous study has demonstrated that the antifungal effect of CA on *F. sambucinum* is achieved through the inhibition of ergosterol biosynthesis (Wei et al., 2020). To further investigate whether CA inhibits ergosterol biosynthesis in *A. fumigatus*, the intracellular ergosterol levels were analyzed in the presence or absence of CA. The results showed no significant difference in ergosterol levels between the CA-treated and untreated groups (Supplementary Figure S1). Since azole resistance in fungi, particularly resistance to ergosterol synthesis inhibition, poses a significant challenge, we aimed to test whether CA exhibits similar antifungal effects against azole-resistant strains of *A. fumigatus*. To evaluate this, we first tested the susceptibility of two itraconazole-resistant strains (R1 and R2) derived from *A. fumigatus* strain Af293 to itraconazole (ITZ). The MICs of R1 and R2 were 8 μg/mL each, which were significantly higher than the MIC of Af293 (1.5 μg/mL) (Figure 2A). Interestingly, the susceptibility of the two itraconazole-resistant strains (R1 and R2) to CA was similar to that of Af293 (Figure 2B). This indicating that CA might not rely on Cyp51A mediated ergosterol biosynthesis inhibition but also has an alternative mechanism of action with the same inhibitory effect on azole-resistant strains.

### Proteomic and metabolomic analyses investigate the molecular mechanisms of CA inhibits *A. fumigatus*

**Figure 3:**
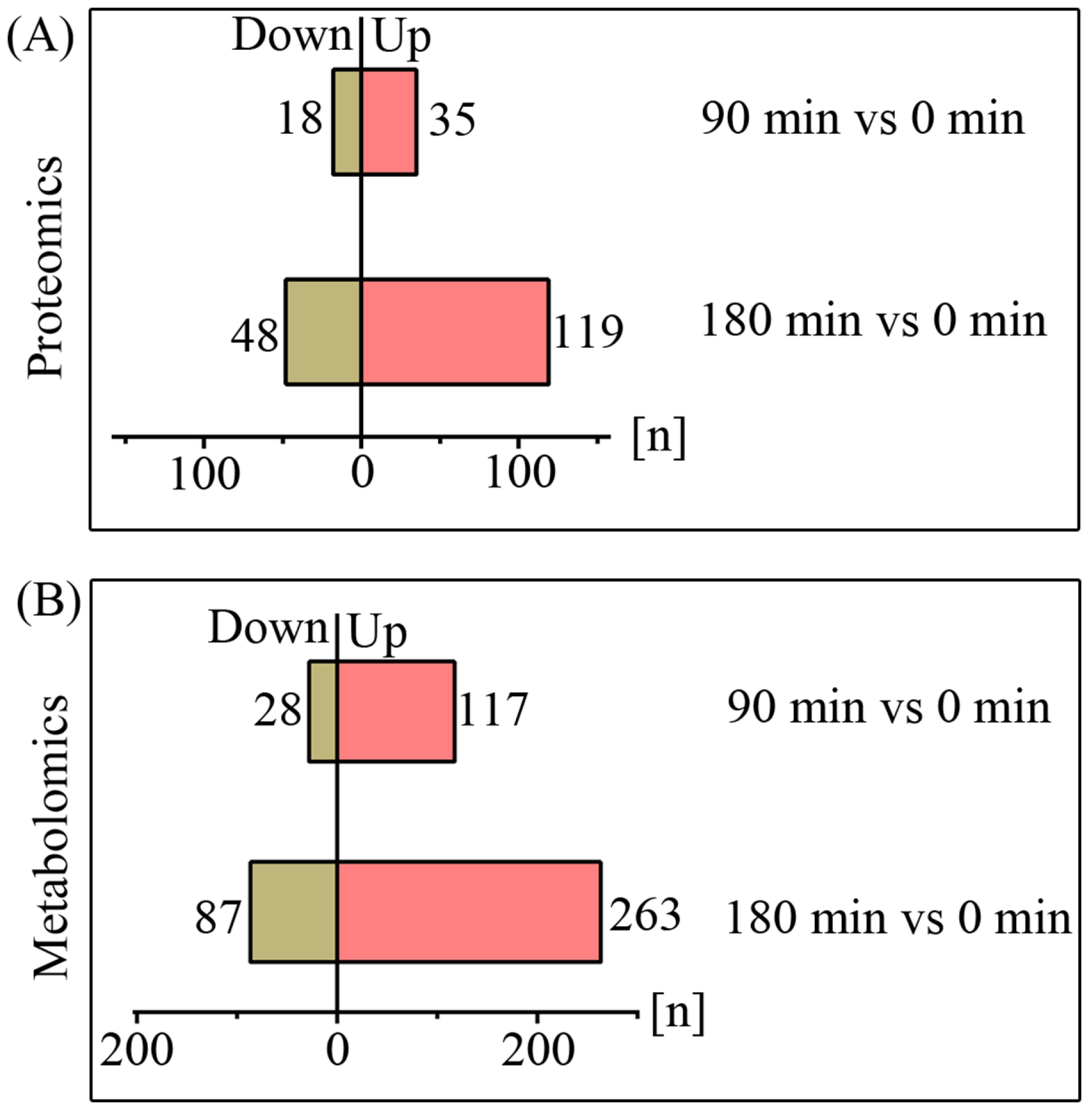
The quantities of differentially expressed proteins **(A)** and metabolites **(B)** upon treatment with CA for 90 and 180 min.

Although some of previous studies have explored the inhibition of fungi by CA, identifying further specific molecular mechanisms will undoubtedly support the development of therapeutic strategies to combat invasive fungal infections (OuYang et al., 2019; Wei et al., 2020; Wang et al., 2024). Here, we employed proteomic and metabolomic analyses to investigate the molecular effects of CA on *A. fumigatus.* The quality control was validated by metabolomic PCA analysis or protein ratio distribution analysis (Supplementary Data Sheet 1 and 2). Proteomic analysis revealed that 53 and 167 differentially expressed proteins (FC > 2 or <0.5, *p* < 0.05) were identified following treatment with CA for 90 min and 180 min, respectively (Figure 3A; Supplementary Table 2). Metabolomic analysis revealed that 145 metabolites showed significant alterations (fold change >2 or <0.5, *p* < 0.05, VIP > 1) after 90 min of CA treatment. Upon extending the treatment duration to 180 min, the number of differential metabolites increased to 350 (Figure 3B; Supplementary Table 3).

### CA disrupt the TCA cycle

**Figure 4:**
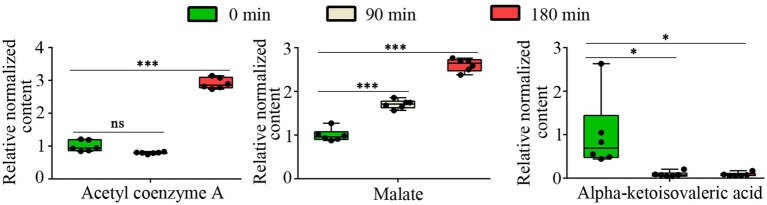
The relative content of Acetyl coenzyme A, malate and alpha-ketoisovaleric acid. “ns” represents no significant difference (*p* value >0.05 or VIP < 1), “*” represents *p* < 0.05 and “***” represents *p* < 0.001.

The TCA cycle is a fundamental metabolic pathway represented in most organisms, playing a crucial role in the oxidation of nutrients and the process of energy production. Malate dehydrogenase (MDH) is an essential enzyme in the TCA cycle that catalyzes the conversion of malate to oxaloacetate. CA has been shown to inhibit the activity of malate dehydrogenase in the TCA cycle of *A. niger* (Wang et al., 2024). Our metabolomic analysis demonstrated that CA treatment induced significant perturbations in tricarboxylic acid (TCA) cycle intermediates of *A. fumigatus*. Specifically, results showed a significant accumulation of malate and acetyl-CoA, while observed a significant decrease in alpha-ketoisovaleric acid following CA treatment, respectively (Figure 4). These metabolic alterations indicate that CA inhibits the growth of *A. fumigatus* by interfering with the tricarboxylic acid (TCA) cycle.

### CA disrupt protein metabolism

**Figure 5:**
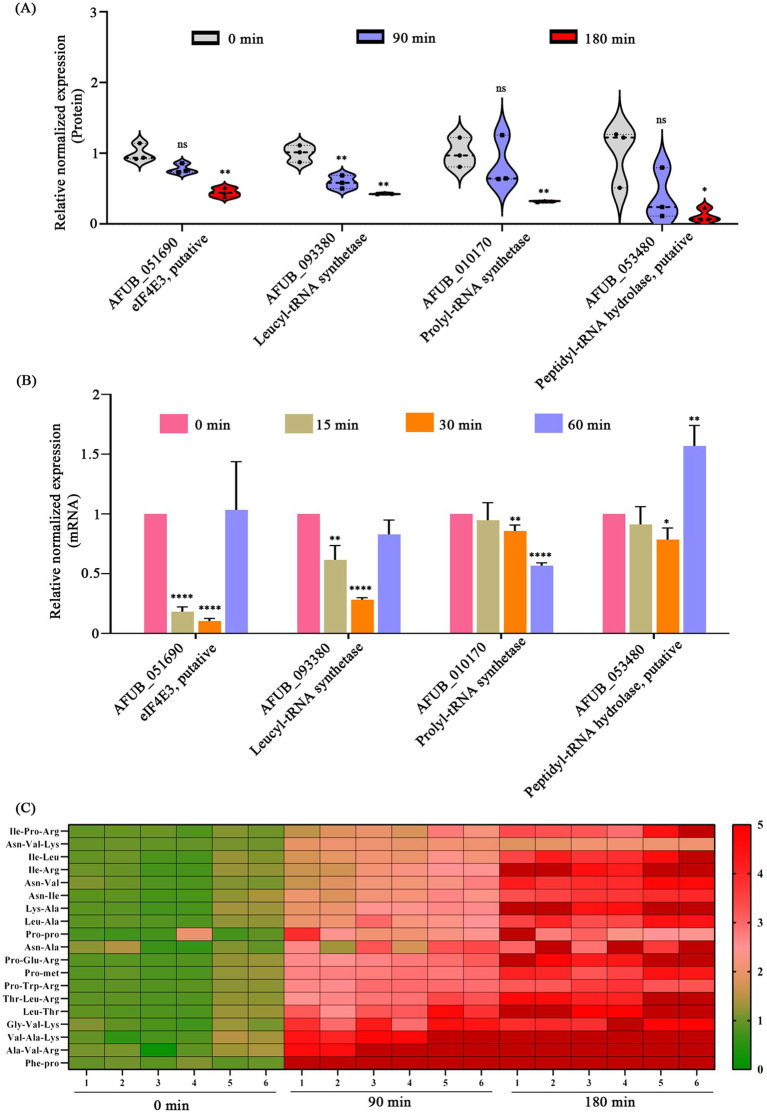
Disruption of protein metabolism by CA. **(A)** Changes in proteins related to protein metabolism in proteomics following CA treatment at 90 and 180 min. **(B)** Validation of mRNA expression levels of protein metabolism-related genes via RT-qPCR after CA treatment at 15, 30, and 60 min. “ns” represents no significant difference, “*” represents *p* < 0.05, “**” represents *p* < 0.01, “***” represents *p* < 0.001, “****” represents *p* < 0.0001. **(C)** The content of intracellular short peptides after treatment with CA for 90 and 180 min (FC > 2 or <0.5, *p* < 0.05, VIP > 1).

Protein group analysis demonstrated that CA treatment led to a significant down-regulation of proteins associated with protein metabolism. Specifically, under the condition of 180-min CA treatment, the putative translation initiation factor eIF4E3 (AFUB_051690), leucyl-tRNA synthetase LeuRS (AFUB_093380), prolyl-tRNA synthetase ProRS (AFUB_0101700), and peptidyl-tRNA hydrolase Pth1 (AFUB_053480) were down-regulated by 2.25, 2.32, 3.13, and 8.33-fold, respectively (Figure 5A). Further, we validated the expression of *eIF4E3*, *leuRS*, *proRS*, and *pth1* at the mRNA level. RT-qPCR analysis revealed that the expression levels of *eIF4E3*, *leuRS*, and *proRS* decreased to varying degrees after 15, 30 or 60 min of CA treatment (Figure 5B). However, the transcription of *pth1* exhibited only a slight decrease following 30 min of CA treatment; conversely, it showed an increase after 60 min of treatment (Figure 5B). Moreover, metabolomic analysis revealed a significant increase in the intracellular accumulation of various short peptides following CA treatment (Figure 5C), supporting the disruption of protein metabolism by CA.

### The putative peptidyl-tRNA hydrolase Pth1 is essential for the growth and CA resistance of *A. fumigatus*

**Figure 6:**
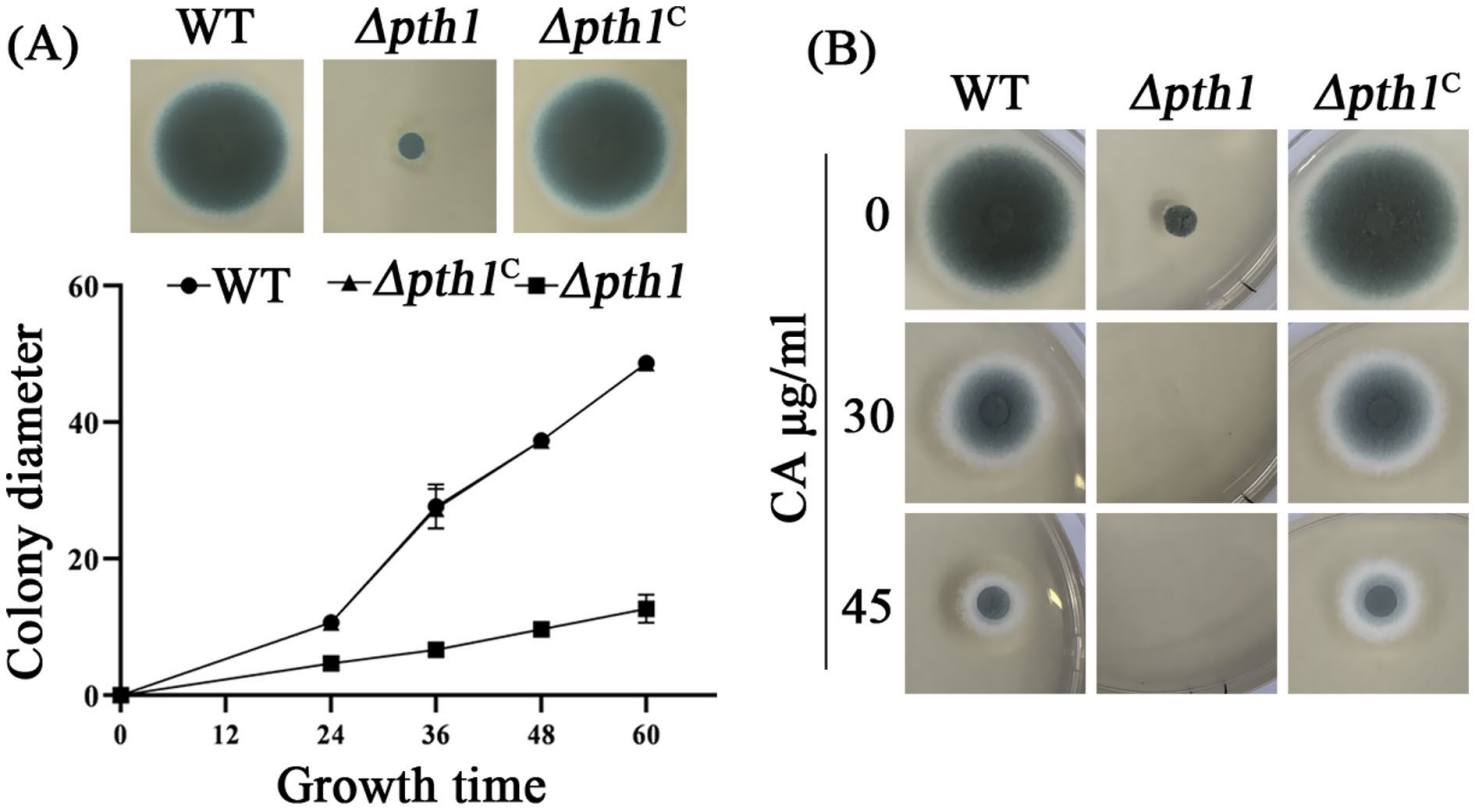
Pth1 is essential for the growth of *A. fumigatus.* **(A)** Growth curve of the *pth1* mutant strain on YUU medium. **(B)** Antifungal sensitivity assay of the *pth1* mutant to CA. Complete absence of growth is seen in the mutated strain, with 30 and 45 μg/mL CA.

Pth1 plays a crucial role in disengaging the nascent polypeptide chain from the tRNA molecule during the process of protein synthesis, which is conserved in both prokaryotes and eukaryotes (Mundra and Kabra, 2024). Although proteomic analysis revealed a significant reduction in Pth1 protein expression following CA treatment (Figure 5A), transcriptional validation of *pth1* via RT-qPCR showed only a minor decrease in mRNA levels after 30 min of CA treatment (Figure 5B). Furthermore, *pth1* transcription was increased after 60 min of CA treatment. To further investigate the function of Pth1 in *A. fumigatus* during its response to CA, we conducted gene knockout analyses of *pth1*using the *A. fumigatus* A1160 strain. Diagnostic PCR analysis showed that *pth1* was completely replaced by the hygromycin resistance gene *hyg*, indicating successful knockout of the *pth1* gene (Supplementary Figure S2). The empirical findings divulged that upon the knockout of *pth1* significantly reduced the growth (Figure 6A), and increased its susceptibility to CA at the concentration of 30 μg/mL CA (Figure 6B). To validate that the growth defect phenotype of *Δpth1* was indeed caused by *pth1* deletion, the *pth1* gene was complemented in the *Δpth1* strain. The complemented strain exhibited growth patterns comparable to the WT, thus confirming that the growth defect in *Δpth1* is attributed to loss of the *pth1* gene (Figures 6A,B). These results suggest that *pth1* is essential for the growth and CA resistance of *A. fumigatus*, and provide valuable insights for further investigations into the molecular mechanism underlying its antifungal effects.

## Discussion

Inhibiting protein synthesis is a well-established target of many antibiotics, including aminoglycosides, tetracyclines, macrolides, and chloramphenicols. Aminoacyl tRNA synthetases play essential roles in the protein synthesis process. They have high specificity and can accurately recognize specific amino acids and tRNA molecules to form aminoacyl-tRNA. This process ensures the correct pairing of amino acids and tRNA, providing accurate substrates for protein synthesis (Kwon et al., 2019). Therefore, aminoacyl tRNA synthetases are an important class of antibacterial targets. At present, three aminoacyl tRNA synthetase inhibitors, mupirocin, tavaborole and halofuginone, have entered clinical practice, which inhibit the functions of isoleucyl-tRNA synthetase (IleRS), leucyl-tRNA synthetase (LeuRS), and ProRS, respectively. Among them, mupirocin and tavaborole can be applied to humans while halofuginone can only be used for veterinary purposes (Bouz and Zitko, 2021). Under conditions of translational stress caused by stalled protein synthesis, misfolded protein accumulation, or nutrient deprivation, Pth1 maintains cellular homeostasis through its dual function of catalyzing peptidyl moiety release from stalled peptidyl-tRNA complexes and preserving the free tRNA pool, making it not only essential for cell survival but also a promising therapeutic target for combating bacterial infections (Mundra and Kabra, 2024).

This study demonstrated that CA disrupts protein metabolism as reflected in three aspects. Firstly, CA significantly suppressed the expression of eIF4E3, LeuRS and ProRS (Figure 5A), key enzymes involved in protein translation and aminoacyl-tRNA synthesis. Secondly, CA treatment led to substantial intracellular accumulation of dipeptides and tripeptides (Figure 5C). Thirdly, *pth1* deletion mutants showed enhanced susceptibility to CA treatment (Figure 6). However, although proteomic analysis revealed a dramatic reduction in Pth1 protein expression following CA treatment for 180 min (Figure 5A), RT-qPCR-based transcriptional validation of *pth1* showed only a minor decrease in mRNA levels at 30 min (Figure 5B). In contrast, after 60 min of CA treatment, the expression of *pth1* increased with statistical significance (Figure 5B). We propose that there may be three reasons accounting for the discrepancy between the proteomic findings and the RT-qPCR verification results regarding Pth1: (1) proteins and mRNA differ in their patterns of expression and also vary in stability; (2) the expression of the Pth1 protein may be regulated at the translational level; (3) there exist methodological differences or experimental errors between proteomics approaches and RT-qPCR.

Mitochondria are essential for the cell growth and survival of the majority of fungi. Hence, mitochondria play a significant role in the drug tolerance and virulence of human fungal infection (Shingu-Vazquez and Traven, 2011; Li and Calderone, 2017). TCA cycle metabolites were primarily regarded as byproducts of cellular metabolism that are essential for the biosynthesis of nucleotides, lipids, and proteins (Martinez-Reyes and Chandel, 2020). Therefore, the TCA cycle represents a potential target for the development of antifungal drugs. Study reported that in *Fusarium solani*, potato glycoside alkaloids have antifungal action via modulating the TCA cycle pathway (Zhang et al., 2024). A study conducted in *Rhizoctonia solani* found that eugenol affected on oxidative phosphorylation and the TCA cycle (Zhao et al., 2021). Isoxanthohumol, extracted from *Humulus lupulus* Linn has been observed to exhibit inhibitory activity against *Botrytis cinerea*. The underlying mechanism involves the suppression of the enzymatic activities of succinate dehydrogenase (SDH) and malate dehydrogenase (MDH), which consequentially results in the perturbation of the TCA cycle (Yan et al., 2021).

The current study revealed that CA treatment significantly enhanced the intracellular accumulation of both malate and acetyl-CoA in *A. fumigatus* (Figure 4). We hypothesize that CA may inhibit key TCA cycle enzymes, particularly malate dehydrogenase (Mdh1) and aconitase (Aco1), given that malate and acetyl-CoA serve as substrates for these respective enzymes. Interestingly, despite these findings, proteomics data revealed no significant changes in the expression levels of the putative malate dehydrogenase Mdh1 (AFUA_6G05210, AFUA_7G05740) and aconitase Aco1 [CDV57_09588 (AFUB_056420)] (Supplementary Table S4), indicating that CA might primarily inhibit the enzymatic activities of these enzymes rather than altering their expression. However, the effects of CA on malate dehydrogenase and aconitase require further experimental validation.

Overall, our study demonstrated that CA disrupts both protein metabolism and TCA cycle in *A. fumigatus*, indicating that it has multiple targets of action. Indeed, relevant studies in other fungi have shown that CA has other antifungal mechanisms, such as inhibiting the activity of ATPase and suppressing the synthesis of ergosterol (Wei et al., 2020; Chen et al., 2023). Since CA is a safe food flavoring additive and approved by the FDA (Friedman, 2017), it holds significant promise for clinical application. However, its poor hydrophilicity, volatility, and decomposition limit its antibacterial activity to a certain extent (Doyle and Stephens, 2019).

Indeed, this study has several limitations. The current study was performed *in vitro*; subsequent research should evaluate the efficacy of CA in animal models of aspergillosis to validate its therapeutic potential. Though metabolomic data indicated a disruption in the TCA cycle, direct enzymatic activity (malate dehydrogenase and aconitase) are strongly recommended to detect under CA treatment. The development of CA derivatives with enhanced stability and bioavailability in future studies could address these challenges, paving the way for novel antifungal treatments. Identifying synergistic combinations (e.g., CA with azoles or echinocandins), characterizing their mutual potentiation mechanisms, and evaluating their efficacy in mouse models of invasive aspergillosis are of interest.

## Data Availability

The mass spectrometry proteomics data presented in this study can be found in online repositories. The names of the repository/repositories and accession number(s) are: ProteomeXchange Consortium/PXD066524.

## Supplementary material

SUPPLEMENTARY FIGURE S1 Relative content of ergosterol content with or without 45 μg/mLCA treatment. “ns” represents no significance.

SUPPLEMENTARY FIGURE S2 Diagnostic PCR for ∆pth1 knockout strain. **(A)** Schematic diagram of *pth1* gene knockout. **(B)** The left homologous arm was amplified using primer pair pth1P1/hyg-de-R, while the right homologous arm was detected with pth1 P6/hyg-de-F primers. No amplification was observed with internal gene primers pth1S1/S2 in ∆*pth1*, whereas fragments of the expected size were produced in the WT strain. These results conclusively demonstrate the complete deletion of the *pth1* gene sequence.
