# Supplementary figures and images for: Antifungal activity of cinnamaldehyde against Aspergillus fumigatus involves disruption of the TCA cycle and protein metabolism
Source: Front Microbiol. 2025 Aug 22;16:1613987. doi: 10.3389/fmicb.2025.1613987 (PMC12411526; doi:10.3389/fmicb.2025.1613987)

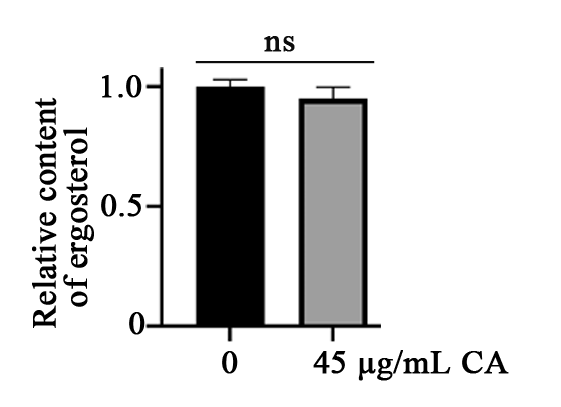

Supplement: SUPPLEMENTARY FIGURE S1 — Relative content of ergosterol content with or without 45 μg/mLCA treatment. “ns” represents no significance. [file Image_1.TIF]

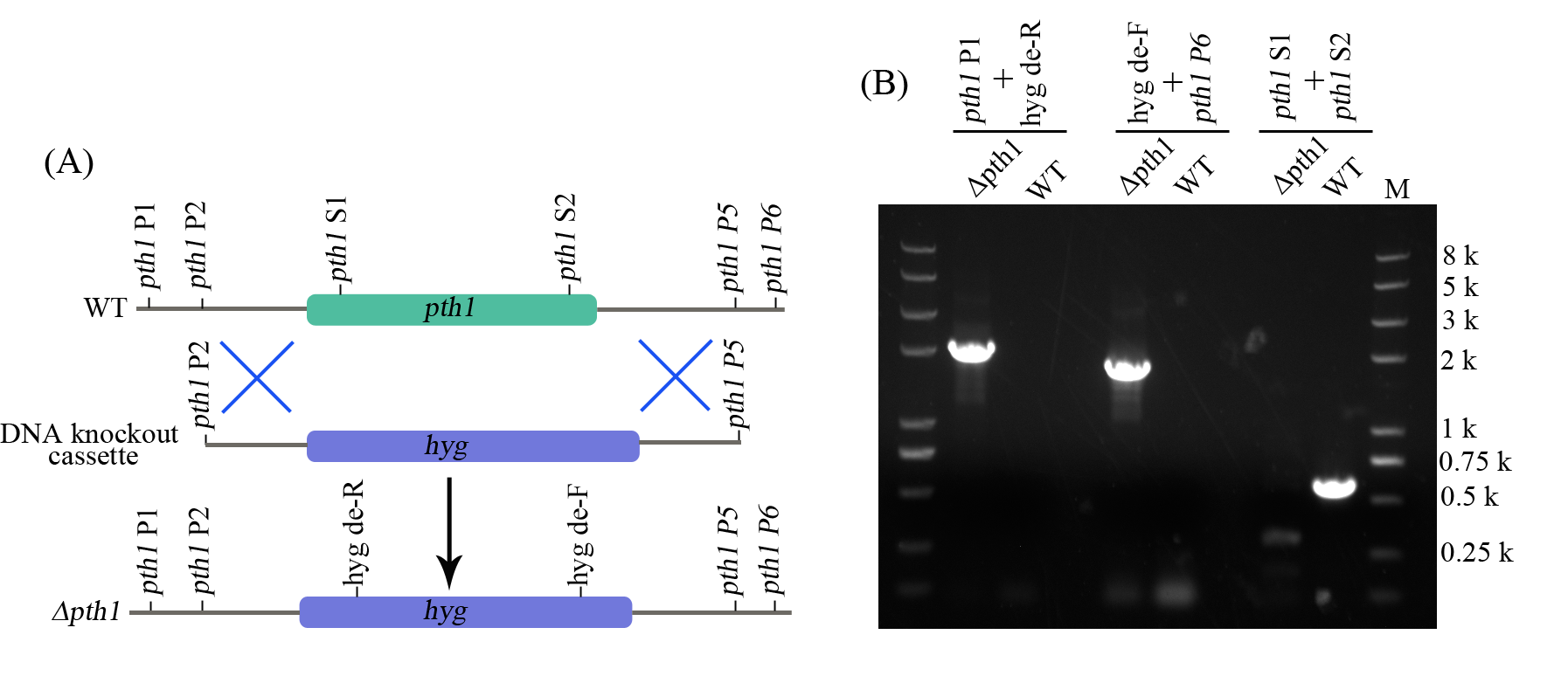

Supplement: SUPPLEMENTARY FIGURE S2 — Diagnostic PCR for ∆pth1 knockout strain. (A) Schematic diagram of pth1 gene knockout. (B) The left homologous arm was amplified using primer pair pth1P1/hyg-de-R, while the right homologous arm was detected with pth1 P6/hyg-de-F primers. No amplification was observed with internal gene primers pth1S1/S2 in ∆pth1, whereas fragments of the expected size were produced in the WT strain. These results conclusively demonstrate the complete deletion of the pth1 gene sequence. [file Image_2.TIF]

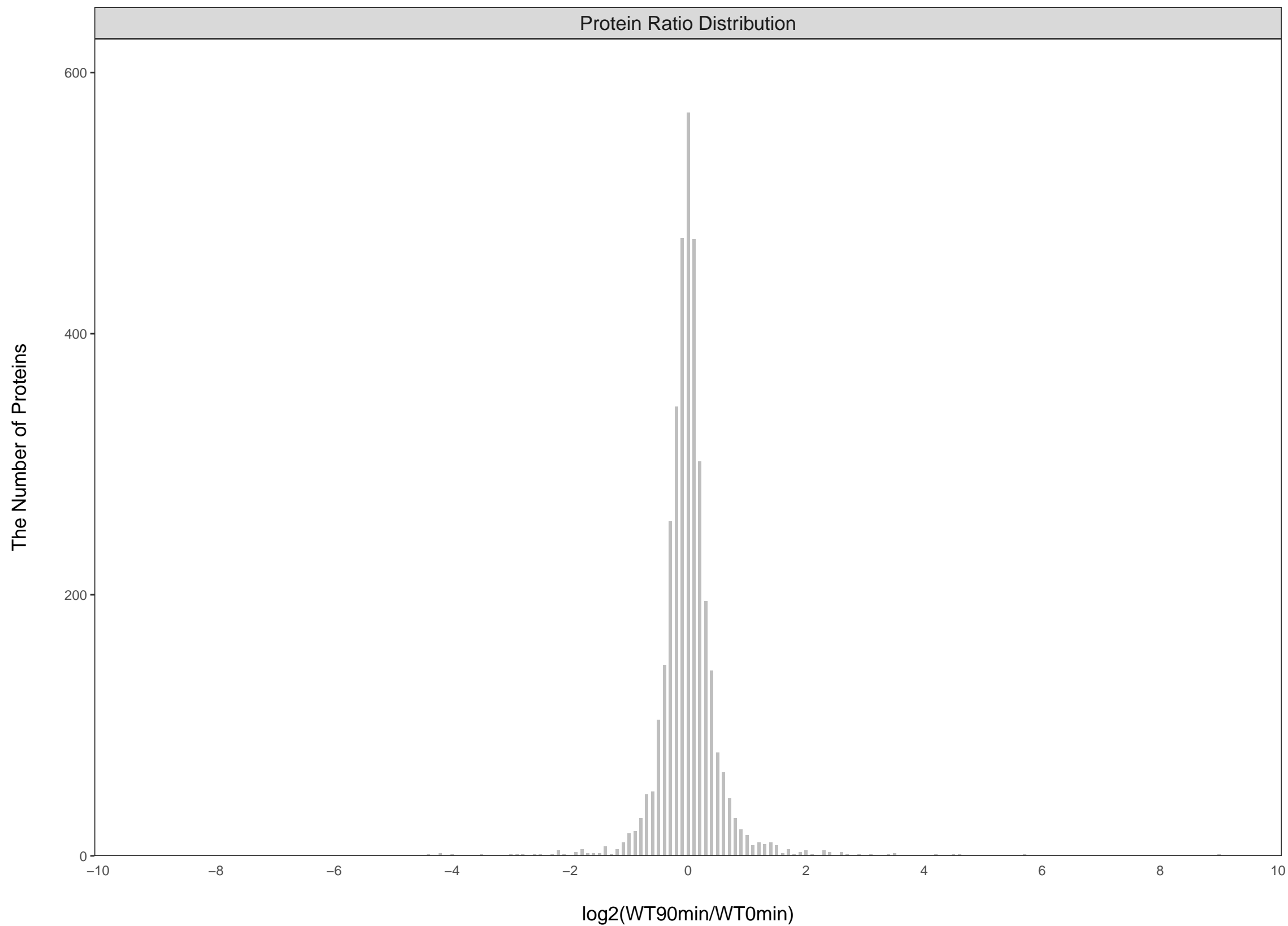

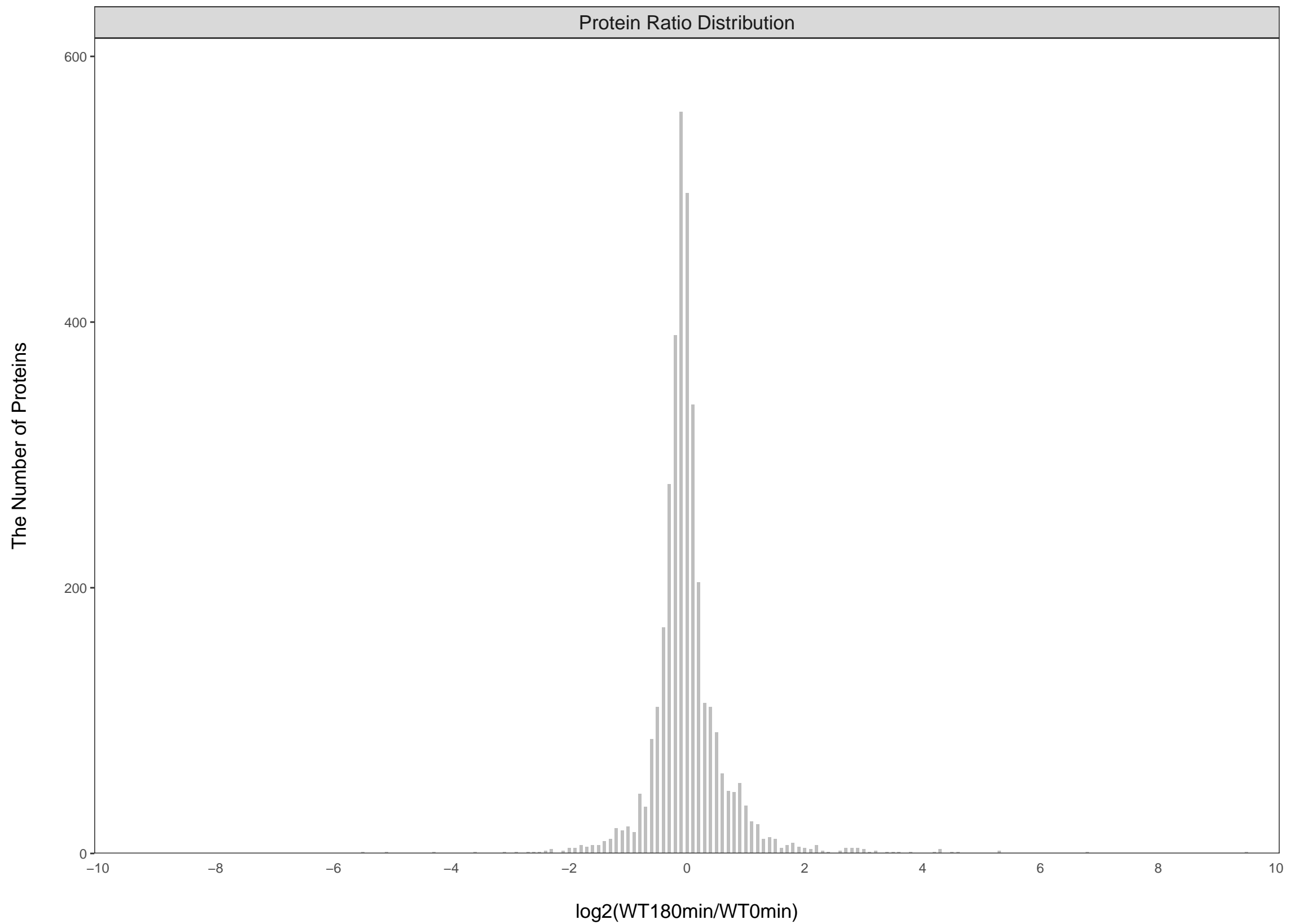

Supplement: Supplementary file 4 [file Data_Sheet_2.pdf]
