# Supplementary material for: Antifungal activity of cinnamaldehyde against Aspergillus fumigatus involves disruption of the TCA cycle and protein metabolism
Source: Front Microbiol. 2025 Aug 22;16:1613987. doi: 10.3389/fmicb.2025.1613987 (PMC12411526; doi:10.3389/fmicb.2025.1613987)
Supplement: Supplementary file 3 [file Data_Sheet_1.pdf]

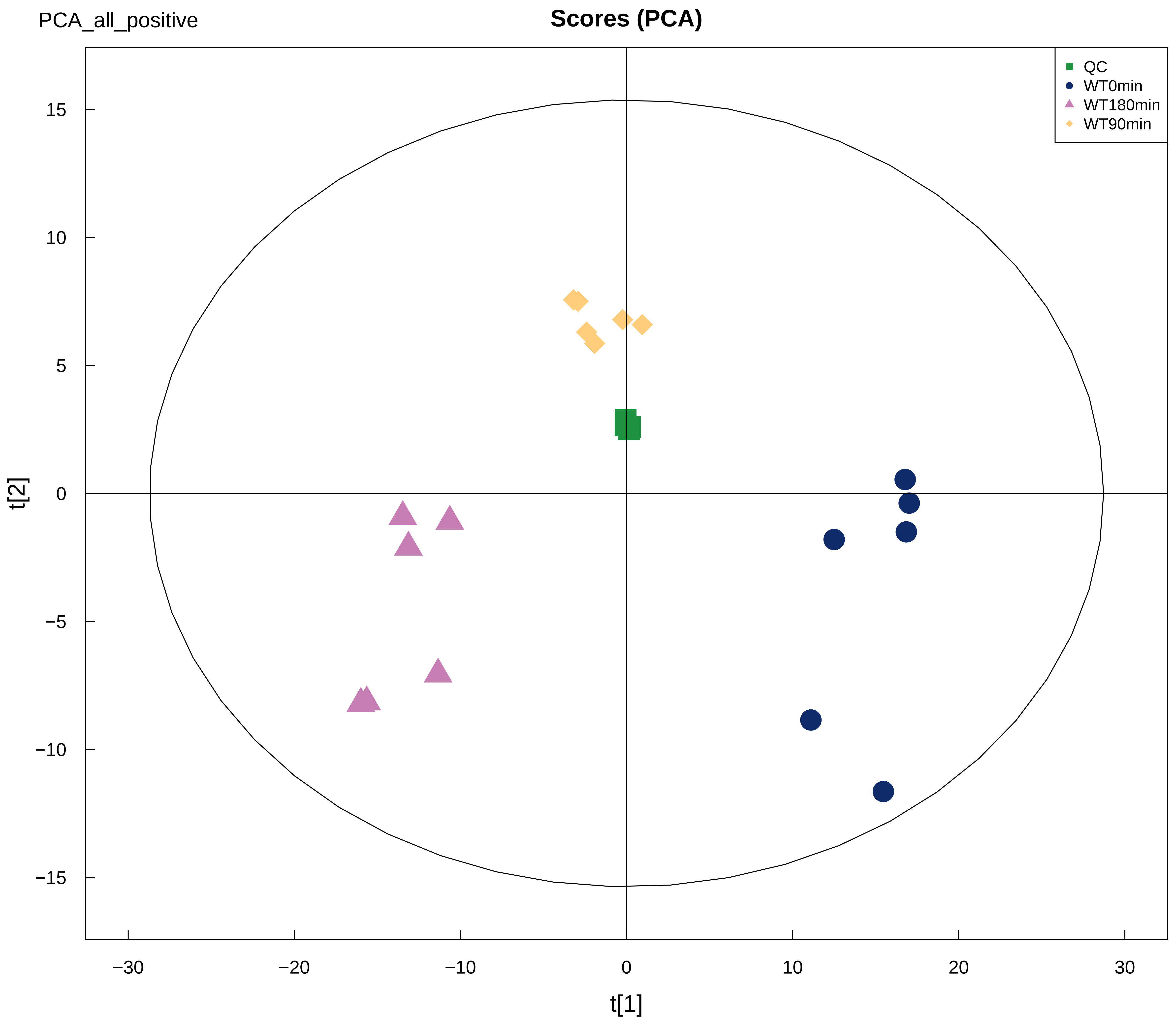
PCA_all_positive
Scores (PCA)
QC
WT0min
WT180min
WT90min
15
10
5
0
−5
−10
−15
t[2]
−30
−20
−10
0
10
20
30
t[1]

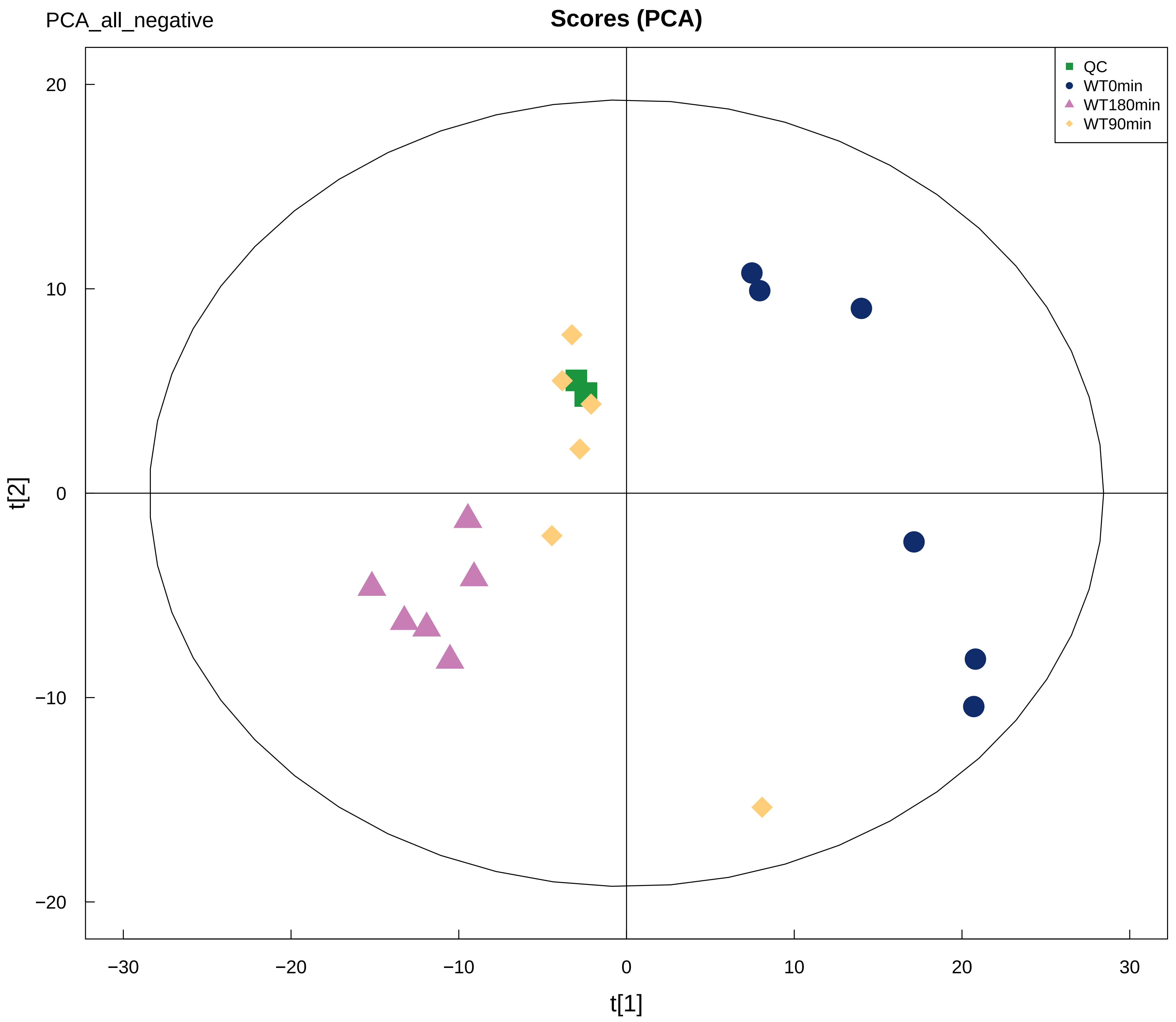

PCA_all_negative
Scores (PCA)
QC
WT0min
WT180min
WT90min
20
10
0
−10
−20
t[2]
−30
−20
−10
0
10
20
30
t[1]
