# Supplementary material for: Antifungal activity of cinnamaldehyde against Aspergillus fumigatus involves disruption of the TCA cycle and protein metabolism
Source: Front Microbiol. 2025 Aug 22;16:1613987. doi: 10.3389/fmicb.2025.1613987 (PMC12411526; doi:10.3389/fmicb.2025.1613987)
Supplement: Supplementary file 5 [file Table_1.docx]

Table S1 Oligonucleotides used in this study

| Primer name | Sequence (5’-3’) |
| --- | --- |
| pth1 P1 | GGATCTTCCAGAGATATCCCTTACCGATCTGTTTCC |
| pth1 P2 | GGAACTATACCAGCGTCGTG |
| pth1 P3 | GCACCGGTCAACCATGATCTTGCGATGCAGGTAACACGG |
| pth1 P4 | CACTCCACATCTCCACTCGATGAAAAATATCCAGCGGGG |
| pth1 P5 | TTGAGGAGCGGTATGAGC |
| pth1 P6 | CTGCCGTTCGACGATATCGCACGTCGCATTGTAGGT |
| pth1 S1 | GTTCCTGTTCATCGCCTCA |
| pth1 S2 | ATCATTGCGGGTTCGTGT |
| hyg F | AGATCATGGTTGACCGGTGC |
| hyg R | TCGAGTGGAGATGTGGAGTG |
| pth1 com F | AACCCTCACTAAAGGGACTAGTCGTGCGGTTATAGTTTCTT |
| pth1com R | CGTTTAAACCTGCAGGACTAGTGAGATTCGGGACCTGAGTA |
| hyg de-F | CTCAAGCCTACAGGACAC |
| hyg de-R | TTTGGACGCCCTACAGAT |
| rt-act1 F | CTGACGGACTACCTGATGAA |
| rt-act1 R | AGAACGCTAGGCTGGAAGAG |
| rt-leuS F | AGCAGGCTGATGTTCTATCC |
| rt-leuS R | TTCCCAGTCGTTCTTCTCG |
| rt-proS F | GATGGGCTGTCATGGGATT |
| rt-proS R | GGATAGCGTCAACGGAAG |
| rt-pth1 F | AAGGTGAGGGTGAAGAGGG |
| rt-pth1 R | ATCATTGCGGGTTCGTGT |
| rt-eIF4E3 F | CACCGTCTCCGACTATCACA |
| rt-eIF4E3 R | CGGGTCCAGACACTCAACAC |
